# Pathology of the “Bridge Cases”

**Published:** 1870-08

**Authors:** Lewis Bauer


Pathology of the "Bridge Cases," by Dr. Lewis Bauer.—In building the Mississippi bridge, at St. Louis, many of the men working at the piers, or beneath them, have been stricken down with paraphlegia and other disorders and symptoms, and four of them have died. Most of the patients, however, have recovered. Those who have died have exhibited evidence of severe inflammation of the brain and spinal cord, and their membranes.

By the manner of construction of the piers the men have been compelled to work in an air-chamber beneath the masonry as the whole was being sunk through the sand to the rock below, the air of which it was necessary to keep at just that pressure which exists in a diving-bell at that depth. The distance below the low water mark which this chamber is forced down, is about 100 feet, 80 of which is through sand; so the workmen have, toward the close of the sinking of each pier, been compelled to labor under a pressure of about 60 pounds to the square inch of surface, or four times greater than normal to the human body.

Paraphlegia is well-marked in every instance, from a slight parasis of motor power to perfect paralysis of both motion and sensation. Numbness usually prevails; notwithstanding this, shooting pains from the spinal down are sometimes felt, but they, with all the hyperaesthesia, wholly cease in the paralyzed limbs.

The spine is always tender; pains in the muscles of the shoulders and chest continue for days after other symptoms disappear. The bladder loses its expulsive power early in the attack, and the urine in a day or two undergoes alkaline decomposition, and often contains blood. On supervention of paraphlegia sensation becomes lost, and the patient may be scalded or burned without pain. Respiration is always disturbed. Previous to death the patient becomes apathetic, stupified, and comatose. The pulse rarely exceeds 105 per minute. A *post mortem* examination in one case showed nearly all the veins dilated and filled with dark tarry blood; great vascularity of the membranes of the cord and brain; a large amount of serum in the arachnoid space. The substance of the brain was changed, and that of the spinal cord, in places, was softened to a pulp, and nearly or quite all the glandular organs showed great vascularity and congestion.

Microscopic examinations showed that the softening of the brain is due, in part at least to inflammatory processes.

The doctor sums up the pathology of these cases substantially, as follows: The great disparity between the pressure of the air within the chamber and the power of the gases and fluids within the body to withstand it, must be a main cause of the trouble. It is certain the equilibrium between these forces is very hard to effect in the short time required in passing into and out of the "chamber," and hence the system would be extremely liable to disaster. Then the quantity of oxygen is proportionately increased in the dense atmosphere beneath the pier, there is hyperoxygenation of the blood, and proportionately increased waste of organic material going on while there. As long as this is going on little injury ensues, but on coming to a rarer atmosphere the power to eliminate this effete organic matter which has accumulated is so reduced that the system suffers from it at once.

I am unable to explain why the lower portion of the spinal cord should be the chief recipient of the poisonous effect."

